# Insufficient Plasma Melatonin and Its Association With Neuropsychiatric Impairments in Patients With T2DM

**DOI:** 10.1155/2024/5661751

**Published:** 2024-07-03

**Authors:** Shuai He, Yue Yu, Peng-quan Chen, Hui-min Sun, Xin-ran Gao, Huai-zhi Sun, Jin-fang Ge

**Affiliations:** ^1^ School of Pharmacy Anhui Medical University, 81 Mei-Shan Road, Hefei 230032, China; ^2^ The Key Laboratory of Anti-Inflammatory and Immune Medicine Ministry of Education Anhui Medical University, Hefei, China; ^3^ Anhui Provincial Laboratory of Inflammatory and Immunity Disease Anhui Institute of Innovative Drugs, Hefei, China; ^4^ Department of Pharmacy North District of The First Affiliated Hospital of Anhui Medical University, Hefei, China

**Keywords:** metabolic syndrome, MLT, neuroinflammation, neuropsychiatric impairments, T2DM

## Abstract

**Purpose:** Type 2 diabetes mellitus (T2DM) is associated with multiple neuropsychiatric impairments, including cognitive dysfunction, and melatonin (MLT) plays a crucial role in maintaining normal neuropsychiatric functions. This study is aimed at investigating the change in plasma MLT levels and its association with neuropsychiatric impairments in T2DM patients.

**Methods:** One hundred twenty-six T2DM patients were recruited, and their demographics and clinical data were collected. Apart from the plasma glycated hemoglobin (HbA1c) levels and other routine metabolic indicators, the plasma concentrations of MLT, C-reactive protein (CRP), Interleukin 6 (IL-6), soluble myeloid triggered receptor 1 (sTREM 1), and receptor 2 (sTREM 2) were measured. Moreover, the executive function and depressive tendency were evaluated via the Behavior Rating Inventory of Executive Function-Adult Version (BRIEF-A) and the Epidemiological Research Center Depression Scale (CES-D), respectively.

**Result:** Compared with the low HbA1c group, the T2DM patients in the high HbA1c group presented lower plasma MLT levels but higher plasma concentrations of inflammatory biomarker levels, together with higher scores in the BRIEF-A and CES-D scales. Moreover, results of the Pearson correlation test showed that the plasma MLT levels were negatively correlated with the BRIEF-A and CES-D scores, as well as plasma concentrations of HbA1c and inflammatory indications, indicating that MLT may mediate their neuroinflammation and neuropsychiatric impairments. Furthermore, the ROC curve results indicated that plasma MLT levels have a predictive effect on executive impairment and depressive status in T2DM patients.

**Conclusion:** MLT levels decreased in patients with T2DM and were associated with neuropsychiatric impairments and inflammatory status, and MLT might be developed as a therapeutic agent and predictive indicator for T2DM-associated executive impairment and depression status.

## 1. Introduction

Type 2 diabetes mellitus (T2DM) diabetes is a metabolic disease characterized by hyperglycemia, constituting a major global health threat as the ninth leading cause of death in the world [1]. In addition to the symptoms of increased blood sugar, insulin resistance, and metabolic disorders brought about by T2DM itself, increasing data have suggested the detrimental effect of T2DM on the structural and functional impairments of the brain, leading to various complications including stroke, cognitive impairment, depression, and even diabetic encephalopathy [2, 3]. It is well known that metabolic disorders of glucose and lipids are risk factors for the dysfunctional changes of the brain, damaging the endothelial cells, resulting in cerebrovascular blockage or rupture, eventually causing morphological and functional changes, accompanied with the declines in attention, memory, and executive functions [4]. Furthermore, insulin resistance is associated with beta-amyloid deposition, which is one of the core neuropathological features in the neurodegenerative disease Alzheimer's disease (AD) [5]. It has been reported that T2DM patients have a significantly increased incidence of cognitive impairment and depression compared to non-T2DM population [6, 7]. Moreover, results of a longitudinal study covering 28,248 middle-aged and elderly people showed that depression, as an independent risk factor, was closely related to the onset of T2DM [8]. In line with these findings, results of our previous studies showed that rats fed with a high-fat diet or challenged with streptozotocin (STZ) presented not only metabolic dysfunction, including hyperglycaemia, hyperlipidemia, and insulin resistance, but also neuropsychiatric impairments, including depression-like behaviors, impaired learning and memory ability, and imbalanced hippocampal synaptic plasticity [9, 10]. These findings suggested again the close connections between T2DM and neuropsychiatric impairments, although the specific mechanism is currently unclear.

Apart from hyperglycemia and insulin resistance, many other risk factors including inflammation [11] have been demonstrated to be attributed to T2DM-associated cognitive dysfunction, directly or indirectly. The insufficient glucose supply in the brain of T2DM could activate the glia, resulting in persistent neuroinflammation through various ways [12], which was supported by the findings in our previous studies that both microglial and astrocyte were activated in the hippocampus of rat injected with STZ, together with an enhanced abundance of inflammatory cytokines including IL-1*β*, Interleukin 6 (IL-6), and TNF-*α* [13]. Consistently, results of clinical studies have demonstrated an increased serum level of IL-6 and C-reactive protein (CRP) in T2DM patients with cognitive impairment, and some inflammatory factors were suggested to be taken as potential biomarkers for predicting T2DM-related dementia and cognitive impairment [14]. Myeloid cell-triggered receptors (TREMs) belong to the rapidly expanding receptor family and are involved in the immune and inflammatory responses of the human body [15]. Besides the role of regulating the process of inflammation and immune response, TREMs have been suggested to play important effect in neuropsychiatric disease including AD [16, 17]. Moreover, the imbalanced abundance of TREM1 and TREM2 has been demonstrated in T2DM patients and the hippocampus and prefrontal cortex of depression rats induced by LPS [18], with a close relationship with not only inflammation indices but also behavioral or neuronal plasticity-associated parameters. These studies suggest that TREM1 and TREM2 may also be involved in neuropsychiatric impairments caused by T2DM.

Melatonin (MLT) is an indole heterocyclic compound produced mainly by the pineal gland and affected by the circadian rhythm; MLT can improve sleep quality and have a strong function in adjusting jetlag [19]. Due to its ability to scavenge free radicals, anti-inflammatory, antioxidant and immunosuppressive, MLT plays a role in a variety of central nervous system diseases [20]. Due to changes in the endocrine system of T2DM patients, the circulating concentrations of various endocrine substances such as adiponectin and leptin, including MLT, have changed, leading to complications in multiple tissues and organs [21, 22]. Decreased abundance of MLT has been found in patients with depression or AD [23], and administration of MLT can alleviate depression and cognitive impairment caused by neuroinflammation through various pathways [24, 25]. Moreover, it has been reported that MLT has a potential effect on diabetes and its complications via regulating insulin secretion [26]. Consistently, in our previous study, we found that MLT can improve both the metabolic dysfunction and impaired learning and memory in T2DM mice induced by a combination of a high-fat diet and STZ intraperitoneal injection, the mechanism of which might be involved with the regulation of circadian rhythm and neuroinflammation [27]. These results suggest that MLT might be a potential therapeutic agent for cognitive impairment caused by T2DM [28].

The purpose of this study is to investigate the change in plasma MLT levels and its association with cognitive impairment in patients with T2DM. T2DM patients were enrolled, and their plasma concentration of MLT and inflammatory markers were measured, together with the routine indicators of glucose and lipid metabolism. The cognitive abilities and depressive symptoms of the patients were also assessed.

## 2. Material and Method

### 2.1. Study Context and Target Population

The target population selected for this study was 126 T2DM patients recruited by the North Endocrinology Department of the First Affiliated Hospital of Anhui Medical University from April 2017 to April 2018. All subjects complied with the Chinese T2DM prevention and treatment guidelines.

### 2.2. Inclusion and Exclusion Criteria

Inclusion criteria include the following: (1) diagnosed as T2DM according to the World Health Organization's 1999 diagnostic criteria; (2) recruiter age ≥ 18 years old); (3) the patient has not received medication treatment recently (at least no treatment of 12 h before blood collected); (4) recruiters have no language communication barriers.

Exclusion criteria include the following: (1) The recruiter has acute complications of diabetes); (2) recruiters with active depression, epilepsy, head injury, or other mental diseases or central nervous system disease); (3) recruiters suffer from serious systemic diseases, such as severe infections and anemia; (4) the recruiter is diagnosed with any substance dependence disease. This research scheme was approved by the Ethics Committee of Anhui Medical University (20170245). All participants provided informed consent forms that comply with the Helsinki Declaration of Principles.

### 2.3. Measurement of MLT Levels and Metabolic Parameters

We measured the height and weight of all participants and calculated their BMI accordingly. The influence of daily energy intake was eliminated by giving patients the same food (provided by the North Canteen of the First Affiliated Hospital of Anhui Medical University). After fasting for 10 h, venous blood samples of the subjects were collected daily from 8:00 a.m. to 9:00 a.m. and then centrifuged at 2500 rpm at 4°C for 20 min. Plasma samples were collected and stored at −80°C for testing. The concentrations of MLT, CRP, IL-6, sTREM1, sTREM2, and insulin were measured using an ELISA kit according to the manufacturer's instructions (MLT: ColorfulGene, Wuhan, China, JYM0697Hu; CRP and IL-6: Huamei Bio, Wuhan, CSB-E08617h, CSB-E04638h, China; sTREM1, sTREM2, and insulin: Meilian Bio, Shanghai, China, ml025411, ml060696, and ml064302). Plasma total cholesterol (TC), triglyceride (TG), high-density lipoprotein (HDL), low-density lipoprotein (LDL), alanine aminotransferase (ALT), aspartate transaminase (AST), total bilirubin (TBIL), direct bilirubin (DBIL), indirect bilirubin (IBIL), serum creatinine (SCR), blood urea nitrogen (BUN), glucose test: fasting blood sugar (FPG), postprandial blood glucose (PBG), and glycated hemoglobin (HbA1c) were measured by biochemistry analyzer and high-performance liquid chromatography–mass spectrometry in the Clinical Laboratory Department of the North district of the First Affiliated Hospital of Anhui Medical University.

According to the 2010 diabetes management guidelines of the American Diabetes Association, plasma HbA1c > 7% is associated with an increased risk of complications of diabetes, especially microvascular complications. Therefore, we divided the subjects into low HbA1c group (HbA1c ≤ 7%) and high HbA1c group (HbA1c > 7%) with a critical value of 7%.

### 2.4. Cognitive Function Assessment

The cognitive function of subjects was assessed using the Behavior Rating Inventory of Executive Function-Adult Version (BRIEF-A). The BRIEF-A scale includes a comprehensive score of behavior regulation index (BRI) and metacognitive index (MI). The BRI includes four items: inhibition, conversion, emotional control, and self-monitoring. The MI includes five items: task initiation, working memory, planning/organization, task monitoring, and material organization. The sum of the scores of each item of the two comprehensive scores is the sum of their comprehensive scores. The sum of all items is the total score of the BRIEF-A scale. All scale items are filled out by the subjects themselves or consulted by professionals. The higher the score, the lower the cognitive function of the subject.

### 2.5. Assessment of Depressive Symptoms

The Epidemiological Research Center Depression Scale (CES-D) was used to evaluate the depressive tendencies of participants. The CES-D scale can be used as a clinical indicator to determine the severity of depressive symptoms and includes 20 items that can measure the frequency and severity of depression. All scale items are filled out by the participants through self-evaluation. The score range of the CES-D scale is 0–60 points, and a higher score indicates a higher level of depression in the subject.

### 2.6. Statistical Analysis

The data was input into EpiData version 3.1, and statistical analysis was performed using SPSS version 17.0 software. Continuous variables with normal distribution were presented as mean ± standard deviation (SD); nonnormal variables were reported as median (interquartile range), and the binary variable is represented as the proportion. Group comparison was carried out using the Student *t*-test, nonparametric test, or chi-square test. Pearson's test was used for correlation analysis, and receiver operating characteristic (ROC) curve analysis was used to determine the cut-off values of area under the curve (AUC) and plasma MLT levels and identify patients with BRIED-A scale T scores > 60 and CES-D scores > 10. *p* < 0.05 was considered statistically significant.

## 3. Results

### 3.1. Comparison of Demography Values and Metabolic Indicators Between Low HbA1c Group and High HbA1c Group

In this study, all subjects were grouped according to the plasma HBA1C level, and their general conditions and routine metabolic indicators were compared. It was found that except for the family history of diabetes, there was no significant difference in the general conditions and metabolic indicators of the two groups of patients.

According to the suggestion that the HbA1c of diabetes patients should not be higher than 7% to avoid complications related to diabetes, the subjects were divided into two groups: low HbA1c group (HbA1c ≤ 7%) and high HbA1c group (HbA1c > 7%). There are 37 people in the low HbA1c group and 89 people in the high HbA1c group. As shown in Table 1, there are no significant differences between the two groups in age, gender, BMI, waist–hip ratio, smoking, drinking, diabetes course, hypertension, and metabolic indicators such as TC, TG, HDLC, LDLC, ALT, AST, TBIL, DBIL, IBI, and insulin. This excludes possible biases caused by other factors of the subjects themselves. However, a significant difference was found between groups with regard to the family history of diabetes, and the probability of patients with low HbA1c having a family history of inheritance is higher.

### 3.2. Increased BRIEF-A Scale Scores in T2DM Patients of High HbA1c Group

We compared and analyzed the cognitive function and depressive tendencies of two groups of patients using the BRAF-A and CES-D scales and found that compared with the low HBA1C group, the high HBA1C group had a decrease in cognitive function and a higher tendency towards depression.

**Figure 1:**
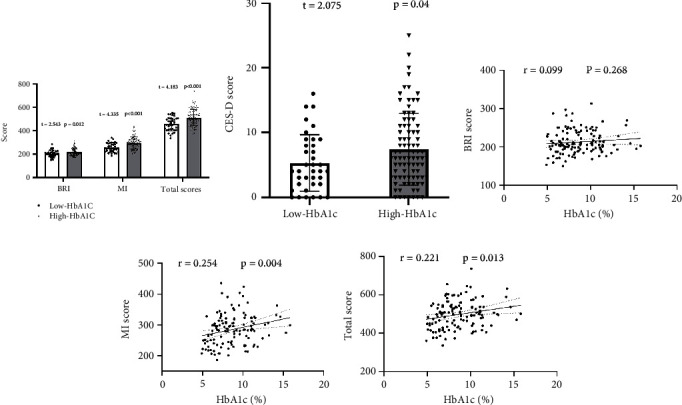
Comparison and correlation of BRI score, MI score, BRIEF-A total score, and CES-D total score between the two groups. (a) Comparison of BRI, MI, and total scores; (b) comparison of CES-D scores; (c–e) correlation between plasma HbA1c levels and three BRIEF-A scores. The number of subjects included in the statistics is 126, with 37 in the low HbA1c group and 89 in the high HbA1c group. Patients in the high HbA1c group have higher total BRI, MI, BRIEF-A scores, and CES-D scores and positively correlated with HbA1c levels, indicating a decrease in cognitive function and an increase in depressive tendencies. Points represent the actual values of each indicator. The dashed lines represent the fitting line. The straight lines represent a regression line.

As demonstrated in Table 2, there was a significant difference in BRIEF-A scores between the two groups of patients. Specifically, in BRI, inhibition and self-control were significantly increased relative to the low HbA1c group (inhibit: *t* = 2.12, *p* = 0.015; self-control: *t* = 2.12, *p* = 0.05). In MI, task initiation, working memory, plan, task monitoring, and organization were higher than that in the low HbA1c group (task initiation: *t* = 4.40, *p* < 0.001; working memory: *t* = 2.79, *p* = 0.006; plan: *t* = 3.98, *p* < 0.001; task monitoring: *t* = 3.84, *p* < 0.001; organization of materials: *t* = 3.06, *p* = 0.001). In addition, there were significant differences in the total BRI score, MI score, BRIEF-A score, and CES-D score between the two groups of patients (Figures 1(a) and 1(b)) (BRI: *t* = 2.54, *p* = 0.012; MI: *t* = 4.34, *p* < 0.001; total score: *t* = 4.18, *p* < 0.001; CES-D total score: *t* = 2.08, *p* = 0.040). Pearson's correlation results showed that the plasma HbA1c levels in TD2M patients were positively correlated with the BRI (*r* = 0.099, *p* = 0.268, Figure 1(c)), MI (*r* = 0.254, *p* = 0.004, Figure 1(d)), and total score of the BRIEF-A scale (*r* = 0.221, *p* = 0.013, Figure 1(e)). This indicates that the executive function and cognitive level of patients in the high HbA1c group are significantly reduced compared to the ones in the low HbA1c group and may be related to their elevated plasma HBA1C levels.

### 3.3. Increased Plasma Concentration of Inflammatory Biomarker Levels in T2DM Patients of High HbA1c Group

To investigate the role of neuroinflammation in T2DM-related neuropsychiatric injury, we compared the levels of four inflammatory markers in the plasma of two groups of patients and found that patients with high HBA1C had higher levels of inflammation.

**Figure 2:**
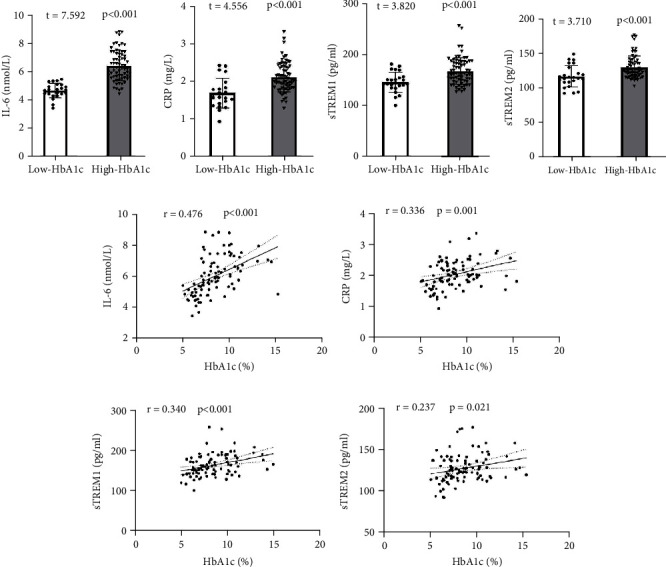
Comparison and correlation of inflammatory markers between two groups of patients. (a–d) Comparison of four inflammatory markers: IL-6, CRP, sTREM1, and sTREM2. (e–h) The correlation between plasma HbA1c and four inflammatory markers: IL-6, CRP, sTREM1, and sTREM2. The number of subjects included in the statistics is 94, with 24 in the low HbA1c group and 70 in the high HbA1c group. The plasma levels of four inflammatory markers increased in patients with high HbA1c and were positively correlated with HbA1c, indicating a stronger level of neuroinflammation.

According to the data in Figure 2, we can see that the plasma concentration of IL-6 (*t* = 7.592, *p* < 0.001, Figure 2(a)), CRP (*t* = 4.541, *p* < 0.001, Figure 2(b)), sTREM1 (*t* = 3.820, *p* < 0.001, Figure 2(c)), and sTREM2 (*t* = 3.710, *p* < 0.001, Figure 2(d)) in the high HbA1c group was significantly increased, as compared to that in the low HbA1c group. In addition, The plasma HbA1c level was positively correlated with the levels of IL-6, CRP, sTREM1, and sTREM2 (Figures 2(e), 2(f), 2(g), and 2(h)), suggesting that compared to the low group of patients, the high group of patients has a higher level of neuroinflammation, which may be related to their plasma HBA1C levels.

### 3.4. Decreased Plasma MLT Levels in T2DM Patients of High HbA1c Group

We explored the plasma MLT levels of two groups of patients and found that the high HBA1C group had lower MLT levels and was negatively correlated with plasma HBA1C levels.

**Figure 3:**
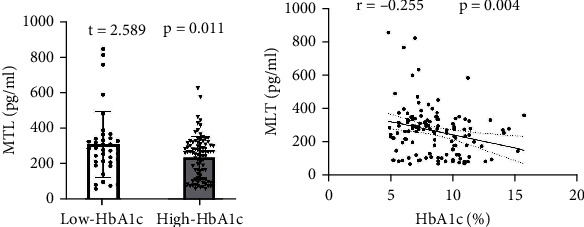
Comparison and correlation of plasma MLT levels between two groups of patients. (a) Comparison of plasma MLT levels; (b) the correlation between plasma HbA1c and MLT levels. The number of subjects included in the statistics is 126, with 37 in the low HbA1c group and 89 in the high HbA1c group. The plasma MLT levels in patients with high HbA1c levels decrease and are negatively correlated with HbA1c levels.

Data in Figure 3 indicates that the plasma MLT concentration was remarkably lower in the high HbA1c group than in the low HbA1c group (*t* = 2.589, *p* = 0.011, Figure 3(a)). In addition, a negative correlation was found between the plasma MLT concentration and HbA1c levels (*r* = −0.255, *p* = 0.004, Figure 3(b)). These results suggested that long-term hyperglycemia may lead to a decrease in MLT secretion and circulating concentration in T2DM patients, and it may be the cause of their neurological and psychiatric damage.

### 3.5. Close Relationship Between Plasma MLT Levels and Metabolic Indicators, Inflammatory Biomarkers, and Scores of BRIEF-A or CES-D Scale in T2DM Patients

Based on the above findings, we conducted correlation analysis on multiple factor variables of T2DM patients to understand their internal structure and relationships.

**Figure 4:**
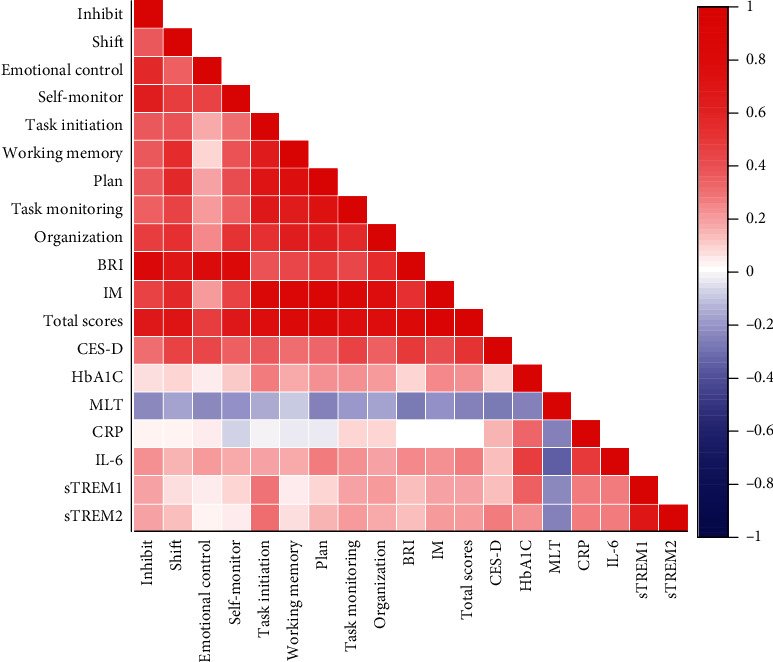
The correlation between BRIEF-A score, plasma HbA1c, MLT, and inflammatory marker levels, in T2DM patients. The red mark indicates a positive correlation between the two, and the blue mark indicates a negative correlation. The depth of the color indicates the size of the correlation coefficient. There are associations between BRIEF-A score, CES-D score, inflammatory markers, and MLT in T2DM patients.

**Figure 5:**
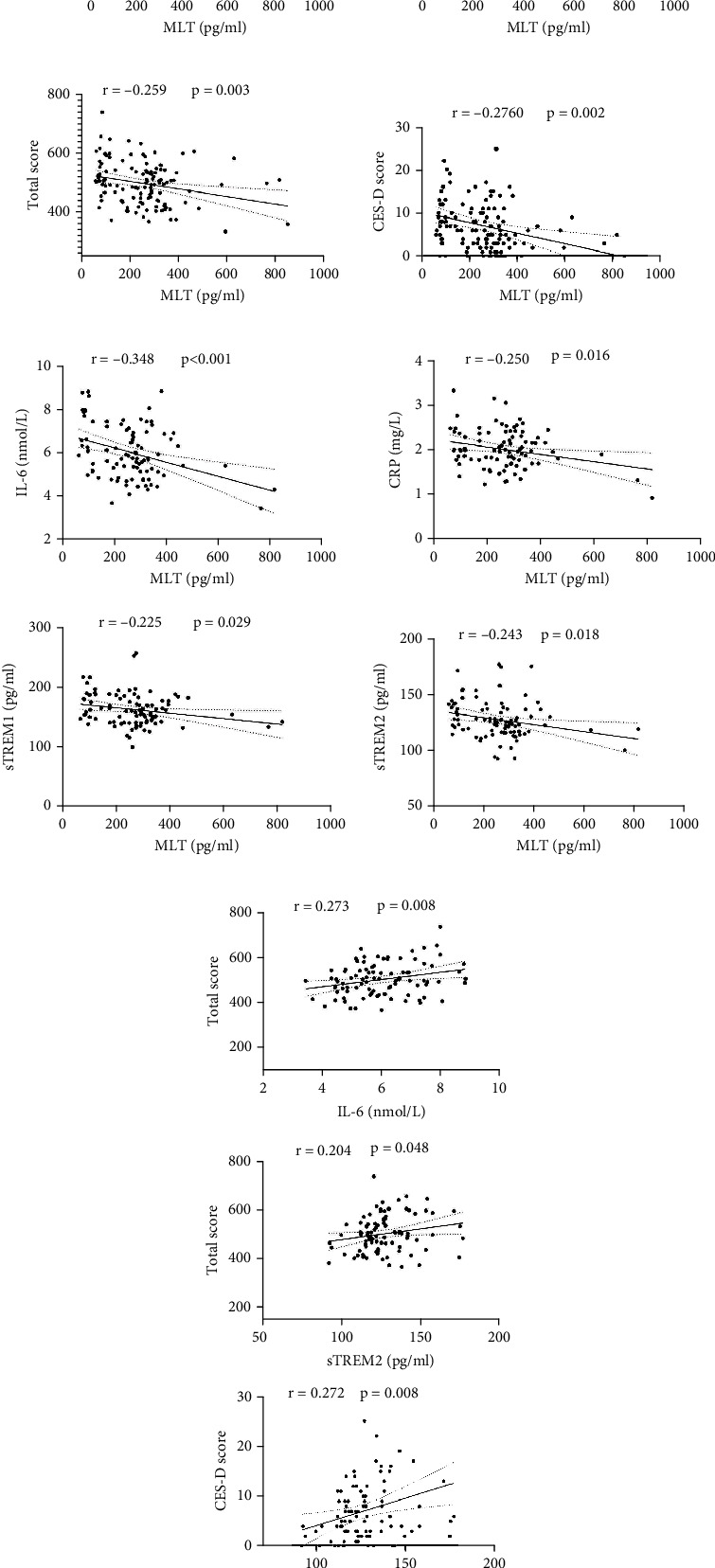
Correlation between BRIEF-A scores, plasma MLT levels, and four inflammatory markers in T2DM patients. (a) The correlation between plasma MLT levels and BRIEF-A and CES-D scores; (b) the correlation between plasma MLT levels and four inflammatory markers; (c) the correlation between IL-6, sTREM2, and the total score of BRIEF-A and CES-D. The number of subjects included in the statistics is 126, with 37 in the low HbA1c group and 89 in the high HbA1c group. The plasma MLT level in T2DM patients is negatively correlated with cognitive function and inflammatory markers, and cognitive function is positively correlated with inflammatory levels.

Figure 4 shows the relationship between plasma HbA1c, MLT, metabolic indicators, and the scores of BRIEF-A and CES-D in T2DM patients, and Figure 5 shows the scatter plot and statistical values of the correlation between plasma MLT, inflammatory markers, and BRIEF-A scale scores in T2DM patients. Among them, there was a negative correlation between the plasma MLT concentration in T2DM patients and the BRIED-A and CES-D scores, as well as the four inflammatory markers IL-6, CRP, sTREM1, and sTREM2 (Figures 5(a) and 5(b)), while the inflammatory markers IL-6 and sTREM2 in T2DM patients were positively correlated with the total score of BRIEF-A and CES-D (Figure 5(c)).

### 3.6. ROC Curve of Plasma MLT Levels in Distinguishing Clinically Significant Executive Function Issues in T2DM Patients

**Figure 6:**
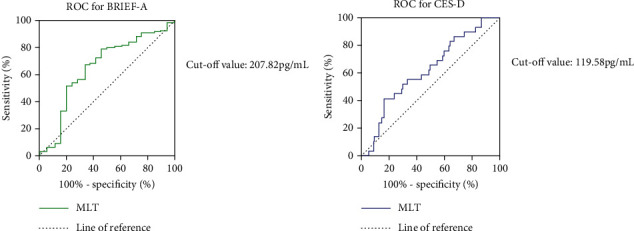
The ROC for MLT grouped by different neuropsychiatric status. ROC curve was used to analyze the diagnostic significance of plasma MLT levels for cognitive impairment and depression status in T2DM patients, by distinguishing samples with *T* scores > 60 or CES-D score > 10. (a) The sensitivity and specificity of the ROC for BRIEF-A were 71.7% and 65.0%, and the cut-off value of MLT was 207.82 pg/mL. (b) The sensitivity and specificity of the ROC for CES-D were 83.5% and 41.4%, and the cut-off value was 119.58 pg/mL.

ROC curve was used to analyze the effect of plasma MLT concentration on the score of BRIEF-A executive function and depression status in T2DM patients. Convert the original BRIEF-A scores of all subjects into T-scores, and T-scores greater than 60 are considered clinically significant executive dysfunction [29]. A CES-D score greater than 10 is considered indicative of possible depressive symptoms. As shown in Figure 6(a), plasma MLT concentration has sensitivity, specificity, and diagnostic value in diagnosing clinically significant executive dysfunction in T2DM patients. The area under the AUC curve is 0.6642 (confidence interval: 0.5245–0.8038), and the sensitivity and specificity were 71.7% and 65.0%, respectively. The critical value of MLT is 207.82 pg/mL. In addition, Figure 6(b) shows the ROC curve results of depression in T2DM patients. The area under the AUC curve is 0.6196 (confidence interval: 0.5068–0.7324), and the sensitivity and specificity were 83.5% and 41.4%. The critical value of MLT is 119.58 pg/mL.

## 4. Discussion

In this study, we investigated the change in plasma MLT levels and explored its association with neuropsychiatric impairments in T2DM patients. Our results showed that the plasma MLT concentration was decreased in T2DM patients with a higher level of HbA1c, and the plasma MLT levels were negatively correlated with not only the BRIEF-A cognitive score and CES-D depression score but also the inflammatory markers such as plasma concentrations of IL-6, CRP, sTREM1, and sTREM2. Moreover, the results of ROC analysis showed a predictive role of MLT concentration in executive dysfunction and depression of T2DM patients. These results indicate that apart from HbA1c, MLT also plays an important role in T2DM-associated metabolic dysfunction and neuropsychiatric impairments, and MLT may serve as a predictive factor for neuropsychiatric impairments in T2DM patients.

Cognitive impairment and dementia are common complications of T2DM, and AD is called Type 3 diabetes in some references (30). Other physiological characteristics shared by these two diseases include inflammation, oxidative stress, and autophagy dysfunction, which are also closely related to brain insulin resistance [31]. It is reported that the duration of diabetes and the high levels of plasma HbA1c are independent risk factors for cognitive impairment in T2DM patients [32, 33], based on which many hypoglycemic drugs are used to treat dementia [34, 35]. Consistently, in the present study, our results showed that the BRIEF-A cognitive function scale scores and CES-D depression scores of patients in the high HbA1C group were significantly higher than those in the low HbA1c group, and there is a positive correlation between plasma HbA1c levels and BRIEF-A cognitive scale scores. Moreover, it has been reported that in T2DM, inflammatory cytokines and chemicals can penetrate the blood-brain barrier and further activate neuroinflammatory responses, disrupting the normal functioning of neurons [36]. In this study, our results showed that the inflammatory markers IL-6, CRP, sTREM1, and sTREM2 were higher in the high HbA1c group than those in the low HbA1c group. Moreover, the plasma HbA1c levels were positively correlated with the levels of inflammatory markers, indicating that neuroinflammation characterized by the release of inflammatory cytokines and activation of myeloid cell-triggered receptors may be involved in the cognitive impairment process of T2DM. Together with the findings in other studies that poor glycemic control in diabetes is related to a greater risk of the development and progression of cognitive impairments [37], these results indicated again that glucose metabolic dysfunction, especially the elevated HbA1c levels, may be a direct factor leading to T2DM-related cognitive impairment. However, the results of the ROC curve did not indicate any predictive effect of HbA1c on the executive dysfunction and depression in T2DM patients in the present study. These results suggested that there might be other factors involved with the neurological and psychiatric changes in T2DM, such as Nesfatin-1 and vitamin D [38, 39].

MLT is an important neuromodulation hormone that participates in not only the regulation of circadian rhythm but also the anti-inflammatory and antioxidant processes in the brain [40, 41]. Acting as an important modulator of glucose production and insulin sensitivity [42], the relationship of MLT with T2DM has been investigated. It has been reported that the polymorphisms of MLT receptor are associated with different glycaemic responses after consuming a diet with high carbohydrates and high glycaemic index levels [43] and might be a genetic candidate for the development of T2DM [44]. Moreover, higher levels of MLT have been demonstrated to be significantly associated with a lower risk of T2DMs [45], and a lower abundance of MLT has been reported in T2DM patients, without or with complications [46, 47]. Targeting its metabolic regulation and neuroprotective effects, our previous results showed that the decline of learning and memory in T2DM model mice could be improved by treatment with MLT. In addition, Xu et al. [48] demonstrated that MLT may have a substantial protective effect on cognition by restoring brain insulin signaling. Although in this study, we cannot compare the difference in serum MLT concentration between T2DM patients and healthy control groups, our research results show that the plasma MLT level of T2DM patients in the high HbA1c group is lower than that of the low HbA1c group, which is consistent with the results reported by Zhang et al. [28] that the serum MLT level of diabetes patients has decreased by 35% compared with the control group, and the serum MLT level of diabetes patients with MCI has decreased by about 15% compared with non-MCI patients. In addition, 24-h serum MLT secretion in patients with diabetes retinopathy is lower than that in patients without diabetes retinopathy [49]. Moreover, our results indicated a negative correlation between plasma MLT levels and the inflammatory markers and the scores of BRIEF-A and CES-D scales. These results suggested that the executive impairment and depression status in T2DM patients may be ascribed, at least partly, to the decrease of plasma MLT levels, and inflammation might be involved with this process. Furthermore, in order to explore the diagnostic significance of plasma MLT levels for cognitive impairment and depression status in T2DM, we conducted ROC analysis on the BRIEF-A cognitive scale and CES-D depression scale scores of subjects. The results showed that a decrease in plasma MLT levels may be not only a risk factor but also a predictive factor for cognitive impairment and depression in T2DM patients, the role which was not found for the HbA1c level. Compared to plasma HbA1c levels, MLT may have higher sensitivity and more accurate predictive value in predicting neuropsychiatric damage in TDM patients.

Of course, this study also has some limitations. Firstly, there were no healthy controls in this study; as a result, we cannot analyze the difference between T2DM and the healthy people. Secondly, the sample size is relatively small, the universality of which should be further explored and extended in large samples. Thirdly, as a cross-sectional study, although we observed changes and correlations in plasma MLT, cognitive function, and inflammatory markers in T2DM patients, we cannot deduce absolutely the specific mechanisms and causal relationships among these factors. Although the therapeutic effect of MLT against T2DM and its associated neuropsychiatric impairments have been carried out in our previous and ongoing animal studies, more in-depth research is needed in the future, focusing on the role and mechanism of MLT in T2DM and its neurological complications.

## 5. Conclusion

In summary, our results suggested a decreased MLT level in T2DM patients, with a close association with the neuropsychiatric impairments and inflammatory status. Moreover, MLT might be developed as a therapeutic drug and predictive indicator for T2DM-associated executive impairment and depression status.

## Figures and Tables

**Table 1:** Comparison of demography values and metabolic indicators between the low HbA1c group and the high HbA1c group.

**Biochemical indicators**	**Low HbA1c group (** **n** = 37**)**	**High HbA1c group (** **n** = 89**)**	**Statistics (** **t**/**Z**/**χ**^2^**)**	**p** **value**
HbA1c (%)	5.127 ± 0.6336	8.667 ± 1.9879	−15.061	< 0.001
Age (year)	54.78 ± 15.30	57.48 ± 13.30	−0.99	0.32
BMI (kg/m^2^)	23.60 ± 3.45	24.79 ± 3.88	−1.61	0.11
WHR	0.91 ± 0.08	0.93 ± 0.06	−1.65	0.1
Gender				
Male	22 (59.46%)	49 (55.06%)	0.21	0.65
Female	15 (40.54%)	40 (44.94%)		
Smoking status				
Smoker	26 (70.27%)	55 (61.80%)	0.82	0.37
Nonsmoker	11 (29.73%)	34 (38.20%)		
Drinking status				
Drinker	25 (67.57%)	51 (57.30%)	1.15	0.28
Nondrinker	12 (32.42%)	38 (42.70%)		
Family history of diabetes				
Have	26 (70.27%)	44 (49.44%)	4.59	**0.03**
None	11 (29.73%)	45 (50.56%)		
Diabetes combining hypertension				
Have	17 (45.95%)	50 (56.18%)	1.1	0.29
None	20 (54.05%)	39 (43.82%)		
Diabetes duration (months)	36 (12; 84)	60 (12; 120)	−1.63	0.10
TC (mmol/L)	4.83 ± 1.91	4.87 ± 1.38	−0.1	0.92
TG (mmol/L)	1.88 ± 2.93	2.14 ± 2.13	−0.57	0.57
HDLC (mmol/L)	0.98 ± 0.40	0.94 ± 0.26	0.73	0.47
LDLC (mmol/L)	3.05 ± 1.33	3.04 ± 1.13	0.03	0.98
ALT (U/L)	26.42 ± 22.10	28.73 ± 28.36	−0.44	0.66
AST (U/L)	20.94 ± 9.24	21.93 ± 13.47	−0.41	0.69
TBIL (*μ*mol/L)	18.12 ± 11.74	16.58 ± 7.92	0.86	0.39
DBIL (*μ*mol/L)	5.14 ± 2.99	4.44 ± 2.98	1.20	0.23
IBIL (*μ*mol/L)	13.24 ± 9.71	12.30 ± 5.51	0.69	0.49
Insulin (m IU/L)	24.63 ± 29.94	23.99 ± 30.35	0.107	0.915

*Note:* The results in bold have statistical significance. Continuous variables with normal distribution were presented as mean ± standard deviation (SD); nonnormal variables were reported as median (interquartile range), and the binary variable is represented as the proportion. Compare the parameters between two groups of patients using the Student *t*-test, nonparametric test, or chi-square test, respectively.

Abbreviations: ALT, alanine aminotransferase; AST, aspartate transaminase; BMI, body mass index; DBIL, direct bilirubin; HDLC, high-density lipoprotein cholesterol; IBIL, indirect bilirubin; LDLC, low-density lipoprotein cholesterol; TBIL, total bilirubin; TC, total cholesterol; TG, triglycerides; WHR, waist-hip ratio.

**Table 2:** Differences in BRIEF-A and CES-D scores between two groups of patients.

**BRIEF-A/CES-D**	**Low-HbA1c group (** **n** = 37**)**	**High-HbA1c group (** **n** = 89**)**	**t** **value**	**p** **value**
Inhibit	48.76 ± 1.17	52.75 ± 1.11	2.12	**0.015**
Shift	53.84 ± 1.56	56.82 ± 0.88	1.76	0.080
Emotional control	51.41 ± 1.69	55.20 ± 1.15	1.82	0.072
Self-monitor	48.57 ± 1.35	52.55 ± 1.09	2.12	**0.050**
Task initiate	50.97 ± 1.74	62.06 ± 1.45	4.40	**< 0.001**
Working memory	60.51 ± 2.10	67.63 ± 1.39	2.79	**< 0.01**
Plan/organization	52.89 ± 1.80	62.54 ± 1.37	3.98	**< 0.001**
Task monitor	49.05 ± 1.13	56.26 ± 1.11	3.84	**< 0.001**
Organization of materials	43.00 ± 1.10	47.92 ± 0.93	3.06	**0.001**
BRI score	202.60 ± 4.65	217.30 ± 3.24	2.54	**0.012**
MI score	256.40 ± 6.41	296.40 ± 5.31	4.34	**< 0.001**
BRIEF-A total score	459.00 ± 9.61	513.70 ± 7.46	4.18	**< 0.001**
CES-D total score	5.27 ± 0.72	7.85 ± 0.74	2.08	**0.040**

*Note:* The results in bold have statistical significance. The number of subjects included in the statistics is 126, with 37 in the low HbA1c group and 89 in the high HbA1c group. Data were presented as mean ± standard deviation (SD), comparing the differences in BRIEF-A score and CES-D score between two groups of patients using the Student *t*-test.

## Data Availability

The dataset generated and/or analyzed during the current research period is not publicly available due to the involvement of patient privacy but can be obtained from the corresponding author (gejinfang@ahmu.edu.cn) upon reasonable request.
